# High Efficacy of Therapeutic Equine Hyperimmune Antibodies Against SARS-CoV-2 Variants of Concern

**DOI:** 10.3389/fmed.2021.735853

**Published:** 2021-09-06

**Authors:** Andres Moreira-Soto, Mauricio Arguedas, Hebleen Brenes, Willem Buján, Eugenia Corrales-Aguilar, Cecilia Díaz, Ann Echeverri, Marietta Flores-Díaz, Aarón Gómez, Andrés Hernández, María Herrera, Guillermo León, Román Macaya, Arne Kühne, José Arturo Molina-Mora, Javier Mora, Alfredo Sanabria, Andrés Sánchez, Laura Sánchez, Álvaro Segura, Eduardo Segura, Daniela Solano, Claudio Soto, Jennifer L. Stynoski, Mariángela Vargas, Mauren Villalta, Chantal B. E. M. Reusken, Christian Drosten, José María Gutiérrez, Alberto Alape-Girón, Jan Felix Drexler

**Affiliations:** ^1^Institute of Virology, Charité-Universitätsmedizin Berlin, Corporate Member of Freie Universität Berlin, Humboldt-Universität zu Berlin, and Berlin Institute of Health, Berlin, Germany; ^2^Centro de Investigación en Enfermedades Tropicales (CIET), Facultad de Microbiología, Universidad de Costa Rica, San Jose, Costa Rica; ^3^Instituto Clodomiro Picado, Facultad de Microbiología, Universidad de Costa Rica, San Jose, Costa Rica; ^4^Instituto Costarricense de Investigación y Enseñanza en Nutrición y Salud, Ministry of Health, Tres Ríos, Costa Rica; ^5^School of Medicine, Universidad de Costa Rica, San Jose, Costa Rica; ^6^Caja Costarricense del Seguro Social, San Jose, Costa Rica; ^7^Centre for Infectious Disease Control, National Institute for Public Health and the Environment, Bilthoven, Netherlands; ^8^German Centre for Infection Research (DZIF), Associated Partner Charité-Universitätsmedizin Berlin, Berlin, Germany

**Keywords:** equine antibodies, SARS-CoV-2, therapy, variant of concern, PRNT titers 50, neutralization test, COVID-19

## Abstract

SARS-CoV-2 variants of concern show reduced neutralization by vaccine-induced and therapeutic monoclonal antibodies; therefore, treatment alternatives are needed. We tested therapeutic equine polyclonal antibodies (pAbs) that are being assessed in clinical trials in Costa Rica against five globally circulating variants of concern: alpha, beta, epsilon, gamma and delta, using plaque reduction neutralization assays. We show that equine pAbs efficiently neutralize the variants of concern, with inhibitory concentrations in the range of 0.146–1.078 μg/mL, which correspond to extremely low concentrations when compared to pAbs doses used in clinical trials. Equine pAbs are an effective, broad coverage, low-cost and a scalable COVID-19 treatment.

SARS-CoV-2 causes coronavirus infectious disease 19 (COVID-19), which leads to either critical illness or death in 5% of patients (1). COVID-19 prevention and treatment options include vaccines, antivirals, and antibody formulations. A wide array of vaccine platforms have shown efficacy in preventing severe disease, but universal access is limited in many resource-limited settings lacking sufficient vaccine coverage (2). Even though there are more than 300 therapeutic drugs in clinical trials, few have proven effective, such as dexamethasone (1, 3). Direct-acting antivirals like Remdesivir are most effective if given very early in the course of the disease, require supplementary oxygen therapy and are very costly at 2,000–3,000 USD per treatment, limiting universal access (4). The use of monoclonal antibodies (mAbs) are safe alternatives shown to enhance viral clearance (5), but their large-scale production is challenging and costly, at around 1,500–6,500 USD per treatment. Polyclonal antibodies (pAbs), either homologous in the case of convalescent plasma and hyperimmune sera, or heterologous such as equine hyperimmune sera, constitute a proven alternative. Convalescent plasma is readily used as COVID-19 therapy due to its rapid capacity of deployment, decade long proven efficacy against emerging diseases such as Ebola and influenza (6), and affordability, at 350–1,000 USD per treatment. Another advantage of convalescent plasma is the use of routine blood donors or follow-up sera of discharged patients, which leads to the production of antibodies against the circulating pathogen, reducing the possibility of immune evasion (6). Nevertheless, patients with mild symptomatology may develop low-titer antibodies as observed for other emerging infectious diseases (6). To overcome this obstacle, hyperimmune globulins can be used, which are prepared from the pooling of many donors. However, both convalescent plasma and hyperimmune sera are donor-dependent, require strict donor rigorous testing for both blood-borne pathogens and high levels of neutralizing anti-SARS-CoV-2 antibodies, all of which might not be readily available in blood bank systems in many developing countries (5, 7). Another low-cost alternative are formulations of intact or fragmented equine polyclonal antibodies (pAbs), widely used for decades as therapies against some viral infections or as antivenoms (8).

We and others have previously shown that horses can be efficiently immunized with different SARS-CoV-2 antigens to yield high quantities of purified pAbs that are 50–80 times more potent than convalescent plasma for virus neutralization (9, 10). A formulation of equine polyclonal F(ab')_2_ fragments against the receptor binding domain (RBD) of SARS-CoV-2 was tested in a multi-center, double-blind, placebo-controlled phase II/III clinical trial showing that it is well tolerated and leads to clinical improvement of hospitalized patients with moderate to severe COVID-19 (11). Additionally, there is an ongoing randomized, multi-center, double-blind, placebo-controlled, dose-finding, phase IIb/III clinical trial (NCT04838821) at hospitals of the Costa Rican Social Security Fund testing equine pAbs formulations to treat moderate and severe COVID-19 cases.

However, pre-clinical data of equine hyperimmune pAbs are only available for early SARS-CoV-2 isolates, whereas such data are lacking for recent and globally circulating variants, considered of concern (VoC) due to their increased transmissibility. VoC alpha, beta, epsilon, gamma and delta (https://www.cdc.gov/coronavirus/2019-ncov/variants/variant-info.html) (lineage designations in Pango/Nextrain: B.1.1.7/501Y.V1 first detected in the United Kingdom, B.1.351/501Y.V2 first detected in South Africa, P.1/501Y.V3 first detected in Brazil/Japan, B.1.427/B.1.429 first detected in the US/California and B.1.617.2/S:478K first detected in India) exhibit a substantial reduction of neutralization by therapeutic mAbs or by antibodies present in the plasma of vaccinated or convalescent individuals (12, 13).

Here we report the results of a plaque reduction neutralization assay (PRNT) against VoC for our purified equine pAbs formulations. PRNT were performed as follows. Briefly, VeroE6 cells (3.25 × 10^5^ cells/ml) were seeded in 24-well plates and incubated overnight. Equine pAbs formulations were mixed in equal parts with a virus solution containing 20 PFU/well previously titrated in the same cells. The experiment was performed in triplicate, and six wells were incubated only with the virus solution containing 20 PFU/well as positive control. The antibody–virus solution was incubated at 37°C for 1 hour and added to the cells. After 1 hour at 37°C, supernatants were discarded, and cells were supplemented with 1.2% Avicel solution in DMEM. After 3 days at 37°C, supernatants were removed, and the 24-well plates were fixed and inactivated using a 6% formaldehyde/PBS solution and stained with crystal violet, and plaques were counted.

The two formulations contain antibodies either against the SARS-CoV-2 recombinant S1 protein (called anti-S1; produced in baculovirus insect cells), or SEM mosaic (called anti-mix; an *E. coli* derived recombinant protein containing the S, E, and M immunodominant regions) derived from the strain Wuhan-Hu-1, Accession Number: YP_009724390 (Native Antigen Company, Oxford, United Kingdom), purified using caprylic acid precipitation method (10). Both formulations effectively neutralized the five VoC and an early isolate of the virus (Germany/Gisaid_EPI_ISL_406862) at similar low inhibitory concentrations (IC_50_ range for anti-S1 formulation: 0.206-1.078 μg/mL; and for the anti-mix formulation: 0.146-0.8359 μg/mL; Supplementary Figure 1; IC_50_ dose-response curves are shown in the Technical Annex). The highest IC_50_ corresponds to the delta VoC (Figure 1). Differences between potencies were statistically significant for the anti-S1 formulation (sum-of-squares F test; *p* < 0.01). This difference was only observed when the delta variant was added to the dataset (sum-of-squares F test without the delta VoC; *p* = 0.9). Previously, the delta VoC has shown resistance to therapeutic monoclonal antibodies due to binding impairment to the spike protein, and fourfold reduced sensitivity in neutralization tests performed in sera of convalescent individuals, suggesting increased immune evasion (14). Therefore, it is expected that a higher dose is needed for neutralization of the delta VoC, in agreement with our data. For the anti-mix formulation, the differences between potencies against tested VoC and early SARS-CoV-2 isolates were not statistically significant (sum-of-squares F test; *p* = 0.3). Notably, IC_50_ values against the five tested VoC of both formulations are extremely low when compared to pAbs doses used by other groups in patients enrolled in clinical trials (4 mg/kg) (11), even at the upper estimates of the 95% confidence intervals, reaching a maximum of 13.89 μg/mL for the beta VoC (Figure 1). Hypothetically, hyperimmunization protocols with potent adjuvants lead to a strong immune response in the horses. Therefore, antibodies likely recognize a diverse variety of epitopes in the viral proteins, showing higher binding affinity that ensure a wider recognition and neutralization than in the case of plasma from vaccinated people or convalescent plasma.

**Figure 1:**
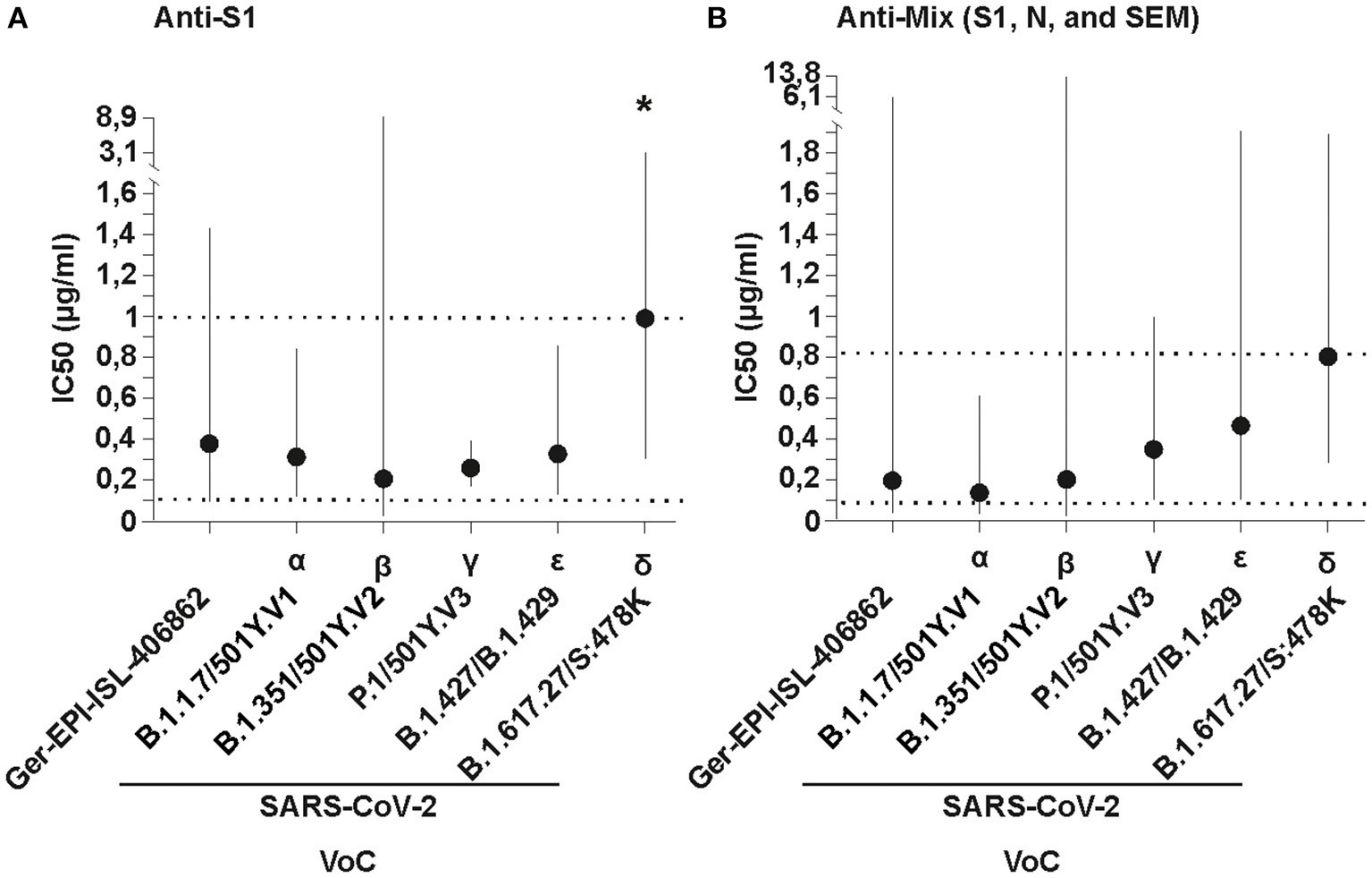
*In vitro* neutralizing potency of **(A)** Anti-S1 (S1 SARS-CoV-2 recombinant protein) and **(B)** Anti-Mix (mixture of S1, N, and SEM mosaic SARS-CoV-2 recombinant proteins of Wuhan-Hu-1, Accession N YP_009724390.1) polyclonal antibodies purified from the plasma of hyperimmunized horses against different SARS-CoV-2 variants of concern (VoC) and an early isolate, named using WHO and Pango/Nextrain designations (strains used = GERMANY/GISAID EPI_ISL 406862, BetaCoV/ChVir21652, hCoV-19/Aruba_11401/2021, hCoV-19/Netherlands/NoordHolland_10915/2021, BetaCoV/ChVir22131/B.1.351/501Y.V2, SARS-CoV-2/CSpecVir25702_4/B.1.617.2 p.1, VS 09.07.2021 acquired from https://www.european-virus-archive.com/evag-news/sars-cov-2-collection). The inhibitory concentration (IC_50_) in plaque reduction neutralization tests (PRNT) was calculated using a non-linear regression analysis in the GraphPadPrism 5 software. Potencies (IC_50_) were not statistically different among viral variants with the Anti-Mix formulation, and the null hypothesis was not rejected, meaning the IC_50_ was equal in all datasets. The potencies (IC_50_) for the Anti-S1 formulations were significantly different, meaning the IC_50_ differed between formulations, but only when the delta VoC was added (denoted by an asterisk). Dotted lines denote the mean minimum and maximum concentrations and vertical solid lines denote 95% confidence intervals for both formulations.

The use of equine pAbs as potential COVID-19 therapy shows several limitations including the need for an early administration during the course of the disease, which is a limitation of all antibody-based therapies, and the risk of adverse reactions to equine immunoglobulins, including serum sickness, owing to the heterologous nature of the preparations. Nevertheless, such reactions can be readily managed pharmacologically, as evidenced by the long-standing experience with the use of equine-derived antivenoms. Our data underscore the high potential of equine pAbs for treatment of COVID-19. As more VoC emerge, further studies should evaluate whether this wide cross-neutralization between SARS-CoV-2 variants is maintained. Shifting antivenom platforms to produce equine pAbs, laboratories in both developed and developing countries that have been manufacturing and distributing safe and standardized antivenoms for decades could rapidly fill the gaps in global demand for therapies that are both effective against VoC and affordable to low- and middle-income countries.

## Data Availability Statement

The raw data supporting the conclusions of this article will be made available by the authors, without undue reservation.

## Author Contributions

AM-S, MA, HB, WB, EC-A, CDí, AE, MF-D, AG, AH, MH, GL, RM, JM-M, JM, ASana, ASánc, LS, ÁS, ES, DS, CS, JS, MVa, MVi, AA-G, JG, and JD conceived, planned and performed the experiments. AM-S and AK carried out the BSL-3 experiments. JD, AA-G, JG, and AM-S planned and carried out the analyses. AM-S, JD, CDr, CR, AA-G, and JG contributed to the interpretation of the results. AM-S, JD, AA-G, and JG took the lead in writing the manuscript. All authors provided critical feedback, helped shape the research, analysis, and manuscript.

## Conflict of Interest

Several authors of this manuscript are employees of Instituto Clodomiro Picado from University of Costa Rica, a public research institute with no commercial interests, where these antibody formulations were developed, and where eventually will be manufactured for use in the Costarican public health system.

## Publisher's Note

All claims expressed in this article are solely those of the authors and do not necessarily represent those of their affiliated organizations, or those of the publisher, the editors and the reviewers. Any product that may be evaluated in this article, or claim that may be made by its manufacturer, is not guaranteed or endorsed by the publisher.

## Supplementary Material

Supplementary Figure 1 IC_50_ dose-response curves of the antibody preparations to SARS-CoV-2 early isolates and variants of concern named using WHO and Pango/Nextrain designations. The Y axis denotes the mean plaque forming units (PFU) per milliliter in triplicate. The X axis denotes the Log10 concentration of the Anti-S1 and the Anti-Mix (combination of S1, N and SEM mosaic protein of Wuhan-Hu-1, Accession N YP_009724390.1) formulations.
