# Supplementary figures and images for: High Efficacy of Therapeutic Equine Hyperimmune Antibodies Against SARS-CoV-2 Variants of Concern
Source: Front Med (Lausanne). 2021 Sep 6;8:735853. doi: 10.3389/fmed.2021.735853 (PMC8451950; doi:10.3389/fmed.2021.735853)

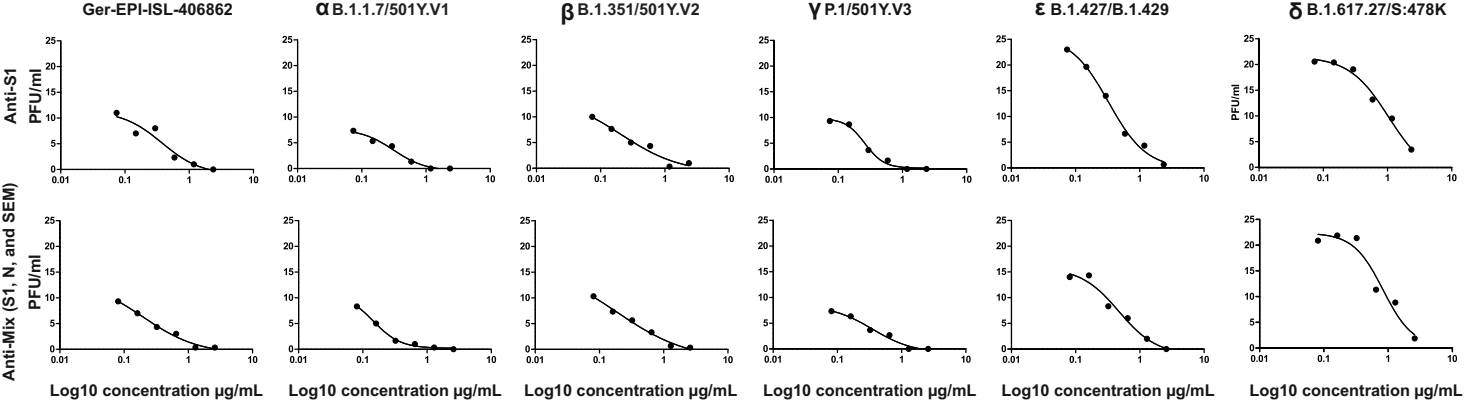

Supplement: Supplementary Figure 1 — IC50 dose-response curves of the antibody preparations to SARS-CoV-2 early isolates and variants of concern named using WHO and Pango/Nextrain designations. The Y axis denotes the mean plaque forming units (PFU) per milliliter in triplicate. The X axis denotes the Log10 concentration of the Anti-S1 and the Anti-Mix (combination of S1, N and SEM mosaic protein of Wuhan-Hu-1, Accession N YP_009724390.1) formulations. [file Presentation_1.pdf]
